# Incidence of RNA viruses infecting taro and tannia in East Africa and molecular characterisation of dasheen mosaic virus isolates

**DOI:** 10.1111/aab.12725

**Published:** 2021-09-07

**Authors:** Dawit B. Kidanemariam, Amit C. Sukal, Adane D. Abraham, Joyce N. Njuguna, Francesca Stomeo, James L. Dale, Anthony P. James, Robert M. Harding

**Affiliations:** ^1^ Centre for Agriculture and the Bioeconomy Queensland University of Technology Brisbane Queensland Australia; ^2^ National Agricultural Biotechnology Research Centre Ethiopian Institute of Agricultural Research Addis Ababa Ethiopia; ^3^ Centre for Pacific Crops and Trees (CePaCT), Land Resources Division (LRD), Pacific Community (SPC) Suva Fiji; ^4^ Department of Biological Sciences and Biotechnology Botswana International University of Science and Technology Palapye Botswana; ^5^ Biosciences Eastern and Central Africa International Livestock Research Institute (BecA‐ILRI) Hub Nairobi Kenya; ^6^ Institute of Molecular Biology and Biotechnology, Foundation for Research and Technology ‐ Hellas Heraklion Greece

**Keywords:** aroids, cucumber mosaic virus, Ethiopia, Kenya, plant viruses, potyvirus, rhabdovirus, Tanzania, Uganda

## Abstract

Taro (*Colocasia esculenta*) and tannia (*Xanthosoma* sp.) plants growing in 25 districts across Ethiopia, Kenya, Tanzania and Uganda were surveyed for four RNA viruses. Leaf samples from 392 plants were tested for cucumber mosaic virus (CMV), dasheen mosaic virus (DsMV), taro vein chlorosis virus (TaVCV) and Colocasia bobone disease‐associated virus (CBDaV) by RT‐PCR. No samples tested positive for TaVCV or CBDaV, while CMV was only detected in three tannia samples with mosaic symptoms from Uganda. DsMV was detected in 40 samples, including 36 out of 171 from Ethiopia, one out of 94 from Uganda and three out of 41 from Tanzania, while none of the 86 samples from Kenya tested positive for any of the four viruses. The complete genomes of nine DsMV isolates from East Africa were cloned and sequenced. Phylogenetic analyses based on the amino acid sequence of the DsMV CP‐coding region revealed two distinct clades. Isolates from Ethiopia were distributed in both clades, while samples from Uganda and Tanzania belong to different clades. Seven possible recombination events were identified from the analysis carried out on the available 15 full‐length DsMV isolates. Nucleotide substitution ratio analysis revealed that all the DsMV genes are under strong negative selection pressure.

## Graphical abstract

Range of symptoms associated with virus infection in taro in East Africa.

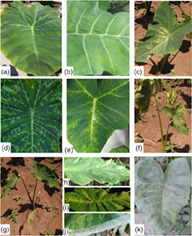

## 1 INTRODUCTION

The aroids, taro (*Colocasia esculenta*) and tannia (*Xanthosoma* sp.) are the most important and widely cultivated edible members of the *Araceae* family in sub‐Saharan Africa (Ndabikunze et al., 2011). In Ethiopia, Kenya, Tanzania and Uganda, taro and tannia are mainly cultivated by small‐holder farmers and play important cultural, economic and nutritional roles (Beyene, 2013; Onwueme & Charles, 1994; Talwana et al., 2009; Tumuhimbise et al., 2009). However, because of various biotic and abiotic factors the yields from taro and tannia in East Africa are much lower than the world's average production (Akwee, Netondo, Kataka, & Palapala, 2015; Talwana et al., 2009; Tumuhimbise et al., 2009). Viruses are among the most economically important pathogens of these crops, resulting in significant yield losses, with a number of viruses reported from different parts of the world (Elliott, Zettler, & Brown, 1997; Revill, Jackson, et al., 2005). The majority of these are RNA viruses, with taro bacilliform virus (TaBV) and taro bacilliform CH virus (TaBCHV), in the genus *Badnavirus*, family *Caulimoviridae* the only DNA viruses reported (Kazmi, Yang, Hong, Wang, & Wang, 2015; Kidanemariam et al., 2018; Yang, Hafner, Dale, & Harding, 2003).

Of the RNA viruses, the potyvirus, dasheen mosaic virus (DsMV, genus *Potyvirus*, family *Potyviridae*) infects aroids wherever they grow (Elliott et al., 1997; Zettler, Foxe, Hartman, Edwardson, & Christie, 1970). DsMV is transmitted in a nonpersistent manner by several aphid species and can also be transmitted by vegetative propagation or sap inoculation (Elliott et al., 1997; Nelson, 2008). The virus has a worldwide distribution and infects both edible and ornamental members of the *Araceae* family (Elliott et al., 1997). Infection typically results in a characteristic feathery mottle and mosaic symptom on the leaves, but symptoms may vary considerably between cultivars and season of the year (Alconero & Zettler, 1971; Elliott et al., 1997). DsMV infection is reported to affect both the quality and quantity of the edible corms, with production losses of more than 25% (Reyes, Rönnberg‐Wästljung, & Nyman, 2006; Valverde et al., 1997).

Taro vein chlorosis virus (TaVCV) is a member of the family *Rhabdoviridae*, genus *Nucleorhabdovirus* (Revill, Trinh, Dale, & Harding, 2005). Typical symptoms associated with TaVCV infection include a distinct vein chlorosis near the leaf margins of infected plants (Pearson, Jackson, Saelea, & Morar, 1999; Revill, Trinh, et al., 2005). TaVCV has been reported from several Pacific Island countries and territories (Atibalentja, Fiafia, Gosai, & Melzer, 2018; Long, Ayin, Li, Hu, & Melzer, 2014; Revill, Jackson, et al., 2005). To date, TaVCV is only known to infect taro, but there is no published information on production losses resulting from infection (Revill, Trinh, et al., 2005). Colocasia bobone disease‐associated virus (CBDaV) is a member of the family *Rhabdoviridae* based on sequence analysis and the presence of characteristic, enveloped, bullet‐shaped particles of ~300 × 50 nm in infected plants (Higgins et al., 2016; Pearson et al., 1999). CBDaV has only been reported from Papua New Guinea (PNG) and the Solomon Islands, where it has been associated with the severe diseases bobone and alomae (Gollifer, Jackson, Dabek, Plumb, & May, 1977; Revill, Jackson, et al., 2005). Bobone disease is thought to be caused by CBDaV alone and is characterised by stunting and gall formation on the pseudostem (Gollifer et al., 1977; Higgins et al., 2016; Pearson et al., 1999; Revill, Jackson, et al., 2005), whereas alomae is a lethal disease usually caused by the dual infection of taro with CBDaV and TaBV (Revill, Jackson, et al., 2005).

Several other viruses have also been reported from aroids worldwide. Taro reovirus (TaRV), a putative member of the genus *Oryzavirus* in the family *Reoviridae*, has been partially characterised based on near full‐length sequences of four different genomic segments of an isolate from PNG (Revill, Jackson, et al., 2005; Revill, Trinh, et al., 2005). However, no symptoms have been associated with TaRV infection and the virus has only been detected in symptomless taro plants and plants infected with other viruses (Revill, Jackson, et al., 2005). Konjac mosaic virus (family *Potyviridae*, genus *Potyvirus*), cucumber mosaic virus (CMV, family *Bromoviridae*, genus *Cucumovirus*), groundnut bud necrosis orthotospovirus (family *Bunyaviridae*, genus *Tospovirus*) and tomato zonate spot virus (tentatively assigned in the genus *Tospovirus*) have also been identified from different aroids (Dong et al., 2008; Manikonda et al., 2011; Sivaprasad, Reddy, Kumar, Reddy, & Gopal, 2011; Wang, Wang, Wang, & Hong, 2014). Of the known viruses reported to infect aroids, DsMV and TaBV are the most widespread (Gollifer et al., 1977; Revill, Jackson, et al., 2005).

We have previously reported the incidence, distribution and molecular characterisation of TaBV and TaBCHV infecting taro and tannia in East Africa (Kidanemariam, Sukal, et al., 2018), but there is no information on the incidence, distribution and diversity of RNA viruses. In this article, we report the results of surveys carried out in 2014 and 2015 to determine the occurrence of four RNA viruses infecting taro and tannia in Ethiopia, Kenya, Tanzania and Uganda. The complete genome sequences and phylogenetic analyses of nine DsMV isolates from East Africa are also reported.

## 2 MATERIALS AND METHODS

### 2.1 Sample collection and nucleic acid extraction

Between November 2014 and June 2015, a total of 171 (160 taro and 11 tannia), 86 (83 taro and three tannia), 41 (29 taro and 12 tannia) and 94 (61 taro and 33 tannia) symptomatic and asymptomatic leaf samples were collected from major growing areas in Ethiopia, Kenya, Tanzania and Uganda, respectively. Leaf samples were desiccated over silica‐gel, transported to the BecA‐ILRI Hub laboratory in Nairobi, Kenya and RNA was extracted (Valderrama‐Cháirez, Cruz‐Hernández, & Paredes‐López, 2002). Following initial screening for viruses at BecA‐ILRI Hub, selected RNA extracts were transported to Queensland University of Technology (QUT), Brisbane, Australia for further analysis.

### 2.2 RT‐PCR, cloning and sequencing

Complementary DNA (cDNA) was synthesised using M‐MuLV reverse transcriptase (Thermo Fisher Scientific, UK) with oligo(dT)_18_ and random hexamers as per the manufacturer's instructions. For the detection of potyviruses and rhabdoviruses, PCR was carried out using published degenerate primers, while virus‐specific primers were used for the specific detection of DsMV, TaVCV, CBDaV and CMV (Table 1). All PCR assays were carried out using 2 μl of cDNA mixed with 10 μl of OneTaq® 2× Master Mix and 5 ρmol of each primer in a total volume of 20 μl. PCR cycling conditions for CBDaV were an initial denaturation of 94°C for 2 min, followed by 35 cycles 94°C for 30 s, 50°C for 30 s and 72°C for 30 s, with a final extension step of 72°C for 5 min. All other PCR assays used published cycling conditions (Table 1). Positive control samples were included for each experiment.

**TABLE 1:** Primers used for virus detection with RT‐PCR

Virus	Primername	Primer sequence (5′‐3′)	Expected size (bp)	Target region	Reference
Potyvirus	CI‐F	GGIVVIGTIGGIWSIGGIAARTCIAC	~700	Cylindrical inclusion protein	Ha et al. (2008)
	CI‐R	ACICCRTTYTCDATDATRTTIGTIGC			
DsMV	DsMV‐3F	ATGACAAACCTGARCAGCGTGAYA	~680	Coat protein	Maino (2003)
	DsMV‐3R	TTYGCAGTGTGCCTYTCAGGT			
CMV	CMV‐F	ATGGACAAATCTGAATCAACC	~780	Coat protein	Wang et al. (2014)
	CMV‐R	TAAGCTGGATGGACAACCCGT			
Rhabdovirus	Rhab‐F	GGATMTGGGGBCATCC	~900	L gene	Dietzgen, Tan, Yong, and Feng (2013)
	Rhab‐R	GTCCABCCYTTTTGYC			
TaVCV	TaVCV‐1	AATATGCTCTCCAGTGTTCACCC	~1,000	L gene	Revill, Trinh, et al. (2005)
	TaVCV‐2	AGGTGCTCAAATGACTCAGCTTGTCC			
CBDaV	CBDV‐3	CTCAAGACAATCAATGGGTGATG	~300	L gene	Ralf Dietzgen, Pers. comm.
	CBDV‐4	CCACGACCGAGTAATTGAC			
Primers used to validate DsMV isolates	U341	CCGGAATTCATGRTITGGTGYATIGAIAAYGG	~900	CP	Yamamoto and Fuji (2008)
	Oligo dT(16)	TTTTTTTTTTTTTTTT			
	NIB‐2F	GTITGYGTIGAYGAYTTYAAYAA	~350	NIb	Zheng, Rodoni, Gibbs, and Gibbs (2010)
	NIB‐3R	TCIACIACIGTIGAIGGYTGNCC			
	HC‐Pro‐F	TGYGAYAAYCARYTIGAYIIIAAYG	~700	HC‐Pro	Ha et al. (2008)
	HC‐Pro‐R	GAICCRWAIGARTCIAIIACRTG			
	DsMV‐1	CACTCATTTGCTCAAATGGC	~970	P1	This study
	DsMV‐2	ACCAACTTTCCATTAAGACG			
	DsMV‐3	CTCTTGCGCGAAGACCCATA	~750	P3	This study
	DsMV‐4	CGCACGACCCCAACCATTTG			
	DsMV‐5	CGAGACCGTAAACTTGGGCG	~700	VPg‐NIa	This study
	DsMV‐6	GTAATTACAATAGAACCATA			
	DsMV‐7	TCCACGAGGATCATGAATTT	~1,000	CI	This study
	DsMV‐8	AAAAATGGAGTTATTTCAAA			
	DsMV‐9	AATCCTCCACCGCCACCACC	~500	CP	This study
	DsMV‐10	ATATAAGCCTCTGCTGCGTC			

PCR products were electrophoresed through 1.5% agarose gels and were stained using GelRed™ (Biotium, USA). Amplicons from representative samples chosen for sequencing were gel‐excised, purified using Freeze ‘N’ Squeeze™ DNA Gel Extraction Spin Columns (Bio‐Rad, Australia), cloned into pGEM®‐T Easy (Promega, Australia) and sequenced using the Big Dye® Terminator v3.1 Cycle Sequencing Kit (Thermo Fisher Scientific, Australia) at the Central Analytical Research Facility, QUT, Brisbane, Australia. For each sample, three independent clones were sequenced with M13F and/or M13R primers.

### 2.3 Generating complete genome sequences of DsMV

Illumina Next Generation Sequencing (NGS) was carried out using selected samples either to generate complete genome sequences of DsMV or identify the presence of other viruses. cDNA libraries were prepared using the Illumina® TruSeq Stranded Total RNA LT Sample Prep Kit with Ribo‐Zero™ Plant, according to the manufacturer's instructions (Illumina, USA). A final concentration of 12 ρmol of pooled cDNA library was sequenced using a 600‐cycles, MiSeqv3 Reagent cartridge (Illumina) and paired‐end reads were generated on the Illumina MiSeq platform at the BecA–ILRI Hub laboratory, Nairobi, Kenya. Subsequently, the NGS data for representative samples were validated by RT‐PCR and Sanger sequencing of cloned PCR amplicons and the 5′‐terminal sequences were obtained by rapid amplification of cDNA ends (RACE) using a 5′/3′ RACE Kit, second generation (Roche, Australia).

### 2.4 Sequence analysis

Sanger‐derived sequences were trimmed to remove primer‐binding sites and analysed using CLC Main Workbench v6.9.2 (Qiagen, USA) and Geneious v11.0.2 (Biomatters, New Zealand). For RNAseq data, adapter sequences were removed using the fastx_clipper and reads were further trimmed to attain optimum quality using the DynamicTrim function of SolexaQA++ v.3.1.3 software (Cox, Peterson, & Biggs, 2010) and fastx‐trimmer module of FASTX‐Toolkit (http://hannonlab.cshl.edu/fastx_toolkit/). De novo assembly of reads from each sample was performed using Trinity v.2.0.3 (Grabherr et al., 2011) and virus contigs were identified by BLASTn analysis against the NCBI‐derived local virus database (ftp://ftp.ncbi.nih.gov/genomes/Viruses/) using a blast command line analysis (Altschul, Gish, Miller, Myers, & Lipman, 1990). Reads were subsequently mapped onto reference sequences using CLC Genomics Workbench v.7.5.1 (https://www.qiagenbioinformatics.com/) and Geneious Prime 2000 (Biomatters) with default parameters. ORFs were predicted and annotated using Geneious Prime 2000 and sequences were designated ‘complete’ based on comparison with the reference sequence used for mapping. Processed Sanger and NGS data were compared to sequences on the NCBI database using BLAST algorithms available on the NCBI website (http://blast.ncbi.nlm.nih.gov/Blast.cgi).

### 2.5 Evolutionary analysis

The evolutionary relationship of East African DsMV isolates with previously reported isolates from the NCBI database was determined through phylogenetic, recombination, selection pressure and pairwise sequence comparison (PASC) analysis. The conserved core coat protein (CP)‐coding region, excluding the heterogeneous N‐terminal sequences, was aligned and analysed using the ClustalW multiple alignment application using BioEdit sequence alignment editor program version 7.1.9 (http://www.mbio.ncsu.edu/BioEdit/bioedit.html). A phylogenetic tree was constructed from ClustalW‐aligned sequences in MEGA7 (http://www.megasoftware.net/mega.php), using the Neighbour‐Joining method with 1,000 bootstrap replications.

To assess for evidence of possible recombination events in DsMV, all available full‐length DsMV sequences were subjected to recombination analysis using the RDP, GENECONV, Bootscan, MaxChi, Chimaera, 3SEQ and SiScan tools embedded in RDP4 (Martin, Murrell, Golden, Khoosal, & Muhire, 2015). Recombination events supported by four or more of the tools (with a Bonferroni‐corrected *p* value of <.05) were considered significant. Breakpoint distribution plot and phylogenetic analysis tools in RDP4 were used to verify the location of individual recombination breakpoints and origin of sequences potentially transferred during each predicted recombination event. Signals identified by RDP4 as potentially being the result of evolutionary processes other than recombination were disregarded. Selective pressures within each distinct protein‐coding region were determined using the average non‐synonymous to synonymous (dN/dS) substitutions ratio in MEGA7.

Pairwise Sequence Comparison (PASC) analysis for DsMV isolates from East Africa together with previously characterised DsMV isolates was carried out on aligned core CP amino acid sequences using Geneious v11.0.2 (Biomatters). In addition, PASC was carried out for each individual protein‐coding region using the nucleotide sequences of the 15 available full‐length DsMV isolates. Pairwise distances of all available full‐length DsMV nucleotide sequences were also assessed using Sequence Distances in the SSE platform (Simmonds, 2012).

## 3 RESULTS

### 3.1 Sample collection and symptoms

**FIGURE 1:**
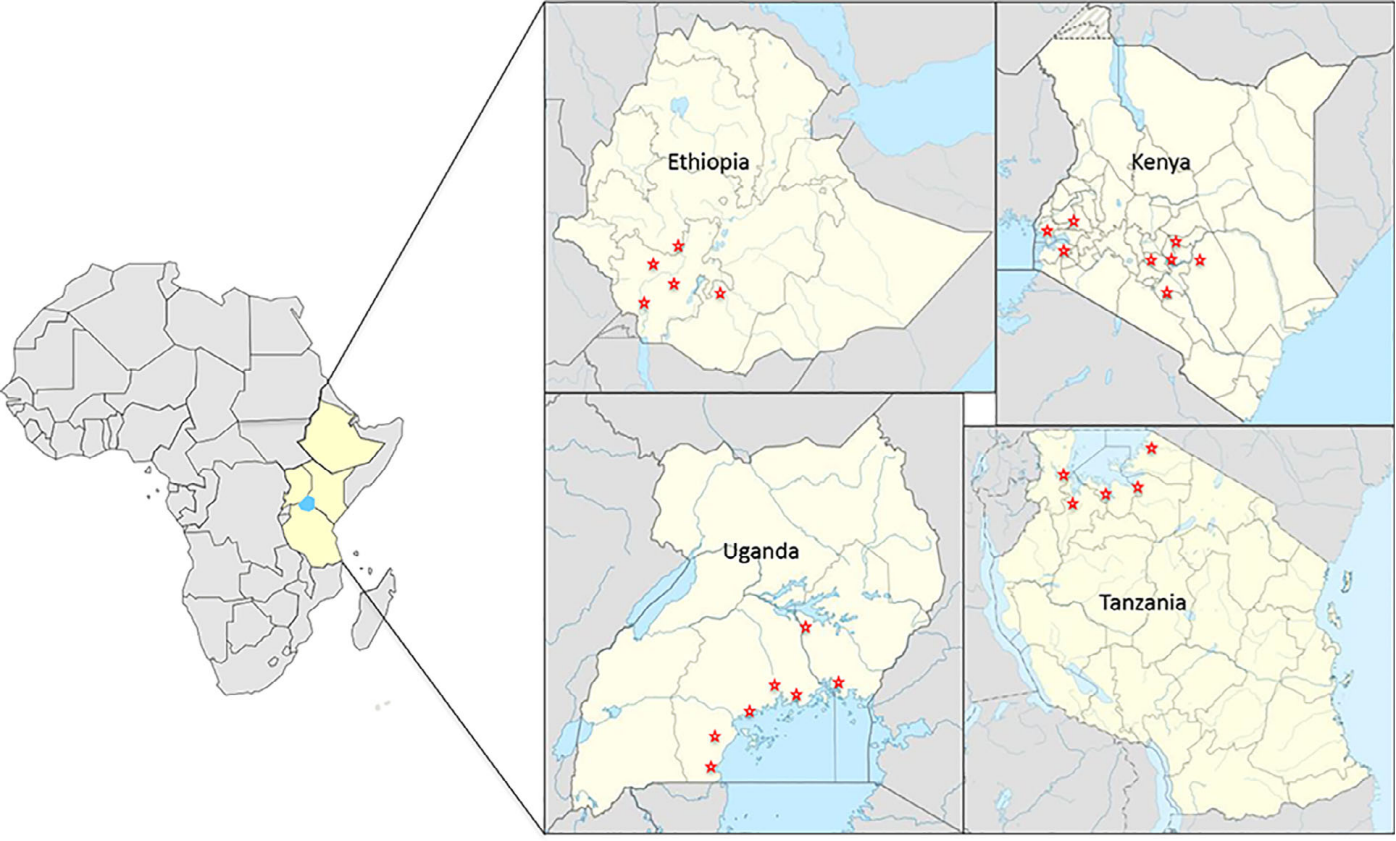
Locations of survey sites in Ethiopia, Kenya, Tanzania and Uganda. Red stars represent sampling sites. A total of 171, 86, 41 and 94 samples were collected from Ethiopia, Kenya, Tanzania and Uganda, respectively

**FIGURE 2:**
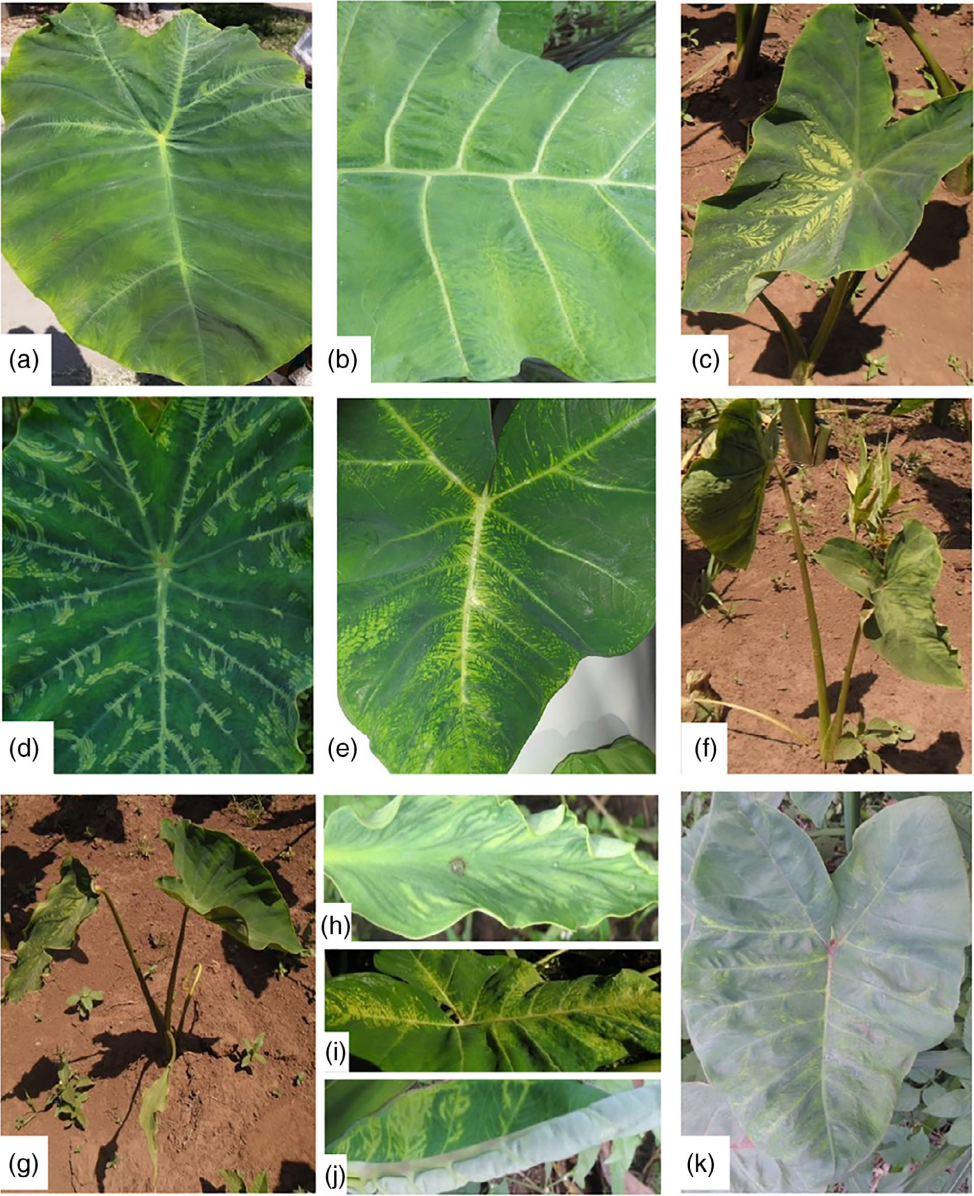
Representative symptoms shown on taro and tannia plants from East Africa. (a) Tz47 showing feathery mottle; (b) Ug31 showing leaf yellowing and vein clearing; (c) Et105 showing feathery mottle and stunting; (d) Et26 showing mosaic and feathery mottle; (e) Et36 showing yellowing and mosaic; (f) Et82 showing mosaic and stunting; (g) and (h) Et51 showing mosaic, leaf distortion, stunting and feathery mottle; (i) Et41 showing yellowing and mosaic; (j) Ug93 showing mosaic; (k) Ug91 showing mosaic and yellowing

Four surveys were conducted covering a total of 25 taro and tannia growing regions of Ethiopia, Kenya, Tanzania and Uganda (Figure 1; Table 2). Of the 392 samples collected, 333 were from taro and the remaining 59 were from tannia, of which 68 taro and 23 tannia plants showed virus‐like symptoms (Figure 2a–k; Table 2). In Ethiopia, taro and tannia plants showing feathery mottle, mosaic, stunting, leaf distortion, leaf yellowing and vein‐clearing symptoms were observed from all regions except Oromia. The highest number of symptomatic samples was collected from Welayita region with 16 out of 87 samples showing virus‐like symptoms. In Kenya, similar virus‐like symptoms were observed on taro growing in all regions except Siaya, whereas in Tanzania, taro or tannia plants exhibiting symptoms were observed in all five locations surveyed. In Uganda, virus‐like symptoms were seen on taro or tannia plants growing at five of the seven regions visited. No plants showing typical alomae or bobone disease symptoms were observed during the surveys.

**TABLE 2:** Summary of virus detection using PCR and RT‐PCR in taro and tannia samples analysed in this study

Country	Region							Number of RT‐PCR positive samples												
		Number of samples collected			Symptomatic samples			Poty			DsMV			CMV^b^	TaVCV	CBDaV	Badnavirus positive samples^a^		Samples infected with both DsMV and badnaviruses	
		Total	Taro	Tannia	Total	Taro	Tannia	Total	Taro	Tannia	Total	Taro	Tannia				Taro	Tannia	Taro	Tannia
Ethiopia	Welayita	87	84	3	16	13	3	17	13	4	17	13	4	0	0	0	75	1	10	3
	Oromia	22	22	0	0	0	0	3	3	0	3	3	0	0	0	0	1	0	1	0
	Sheka	25	22	3	6	4	2	9	5	4	9	5	4	0	0	0	7	3	2	2
	Masha	14	12	2	3	1	2	4	2	2	4	2	2	0	0	0	3	1	2	0
	Keffa	23	20	3	4	1	3	3	1	2	3	1	2	0	0	0	6	3	1	2
	Total	171	160	11	29	19	10	36	24	12	36	24	12	0	0	0	92	8	16	7
Kenya	Nyeri	30	29	1	9	9	0	0	0	0	0	0	0	0	0	0	17	0	0	0
	Laikipia	3	2	1	1	1	0	0	0	0	0	0	0	0	0	0	1	0	0	0
	Tharaka Nithi	14	14	0	8	8	0	0	0	0	0	0	0	0	0	0	10	0	0	0
	Kirinyaga	9	8	1	3	3	0	0	0	0	0	0	0	0	0	0	5	0	0	0
	Embu	19	19	0	4	4	0	0	0	0	0	0	0	0	0	0	13	0	0	0
	Kakamega	4	4	0	1	1	0	0	0	0	0	0	0	0	0	0	4	0	0	0
	Kisumu	5	5	0	1	1	0	0	0	0	0	0	0	0	0	0	3	0	0	0
	Siaya	2	2	0	0	0	0	0	0	0	0	0	0	0	0	0	1	0	0	0
	Total	86	83	3	27	27	0	0	0	0	0	0	0	0	0	0	54	0	0	0
Tanzania	Musoma	9	9	0	2	2	0	0	0	0	0	0	0	0	0	0	3	0	0	0
	Tarime	5	2	3	1	0	1	0	0	0	0	0	0	0	0	0	1	3	0	0
	Mago	2	2	0	1	1	0	0	0	0	0	0	0	0	0	0	2	0	0	0
	Biharamulo	9	1	8	1	0	1	2	0	2	2	0	2	0	0	0	0	8	0	1
	Mwanza	16	15	1	9	7	2	1	1	0	1	1	0	0	0	0	7	1	1	0
	Total	41	29	12	14	10	4	3	1	2	3	1	2	0	0	0	13	12	1	1
Uganda	Busuju	25	16	9	9	5	4	0	0	0	0	0	0	0	0	0	15	4	0	0
	Lukaaya	26	17	9	4	4	0	1	0	1	1	0	1	0	0	0	15	5	0	0
	Busiro	20	11	9	3	1	2	0	0	0	0	0	0	0	0	0	10	1	0	0
	Budondo	4	4	0	0	0	0	0	0	0	0	0	0	0	0	0	4	0	0	0
	Buunya	6	5	1	1	1	0	0	0	0	0	0	0	0	0	0	5	0	0	0
	Kignlu	3	2	1	0	0	0	0	0	0	0	0	0	0	0	0	2	1	0	0
	Luuka	10	6	4	4	1	3	0	0	0	0	0	0	3	0	0	5	3	0	0
	Total	94	61	33	21	12	9	1	0	1	1	0	1	3	0	0	56	14	0	0

^a^ Data from Kidanemariam, Sukal, et al. (2018).

^b^ All three samples testing positive for CMV from Uganda are from tannia. Data from Kidanemariam et al. (2019).

### 3.2 RT‐PCR screening

When RNA extracts were tested for the presence of potyviruses by RT‐PCR using the degenerate primers, CI‐F/R, the expected ~700 bp amplicon was observed in 36 (24 taro and 12 tannia) samples from Ethiopia, as well as one sample from Uganda (Ug31, Lukaaya region) and three samples from Tanzania (Tz24 and Tz34 from Biharamulo and Tz47 from Mwanza). Samples Ug31, Tz24 and Tz34 were from tannia, while Tz47 was from taro. When these 40 samples were subsequently tested for DsMV by RT‐PCR using specific primers DsMV‐3F/3R, the expected amplicon of ~560 bp was obtained from all 40 samples.

Testing of the extracts for the presence of CMV using the specific primers, CMV‐F/R targeting the CP‐coding region, resulted in an amplicon of the expected size from only three tannia samples (Ug90, 91, 92) from Buikwe district in Uganda. The amplicons from the three samples were cloned and sequenced, with BLAST analysis of the trimmed 735 bp region of the cloned sequences revealing the highest identity (96%) to a subgroup IB CMV isolate (HM3) from Egypt (GenBank accession number KX014666).

When extracts were tested for the presence of rhabdoviruses using the degenerate primers RhabF/R, the expected ~900 bp product was generated from 13 samples. However, these samples all tested negative for TaVCV and CBDaV using virus‐specific primers, despite amplicons of the expected size (~220 and ~700 bp, respectively) being generated from the positive controls. Subsequent sequence analysis of cloned amplicons generated using the degenerate rhabdovirus primers revealed that the sequences were of chloroplast origin.

### 3.3 Sequence analysis

Following RT‐PCR using the degenerate potyvirus primers, amplicons from five samples selected from different locations (Et9, Et41, Et56, Tz34 and Ug31) were cloned and sequenced. BLAST analysis of the trimmed 630 bp sequences revealed 79–89% and 90–99% identity at the nucleotide and amino acid levels, respectively, to DsMV isolates infecting either taro from India (Et41, Tz34 and Ug31) or *Zantedeschia aethiopica* (arum lily) from China (Et9 and 56). Amplicons generated using the DsMV‐specific primers from 16 representative samples were subsequently cloned and sequenced. These 16 samples included 13 from Ethiopia (Et5, 9, 26, 29, 36, 40, 41, 51, 56, 74, 82, 105, 106), as well as samples Tz24 and 34 from Tanzania and sample Ug31 from Uganda. BLAST analysis of the trimmed 520 bp sequences from the 16 samples revealed a maximum of 92–96% and 98–99% identity at the nucleotide and amino acid levels, respectively, to DsMV isolates infecting a range of aroids from China, Japan, India and Nicaragua.

Following the analysis of these partial sequences, the complete genome sequences of DsMV isolates Ug31, Tz34 and seven isolates from Ethiopia (Et5, 9, 26, 29, 36, 41 and 56) were generated using Illumina MiSeq NGS (Table S1). A further 15 samples were also subjected to NGS, including Ug90, 91 and 92, which were positive for CMV and 12 samples where no viruses were identified by RT‐PCR (Table S2). Of these, four samples (Ke11, Ke63, Tz2 and Tz24) were asymptomatic, while eight samples displayed some virus‐like symptoms (Et39, Ke23, Ke28, Tz22, Tz25, Ug35, Ug44 and Ug70).

For the nine DsMV samples, raw reads totalling between 1,164,718 and 2,855,123 were obtained, which after trimming resulted in between 971,694 and 2,296,872 reads per sample (Table S1). Between 202,476 and 426,072 reads aligned to the reference sequence. Comparison of the consensus nucleotide sequences of the nine isolates derived from NGS with the respective consensus RT‐PCR‐generated CP sequences revealed 99–100% identity. The complete genome sequences of the nine DsMV isolates varied from 10,018 to 10,028 nucleotides in length, excluding the 3′ poly(A)‐tail. The 5′ and 3′ UTRs of all the isolates varied between 166–168 nucleotides and 239–249 nucleotides, respectively. The genome sequence for all the isolates contained a single large ORF of 9,612 nucleotides, encoding a predicted polyprotein of 3,204 amino acids, with predicted molecular masses of 364 kDa. Furthermore, the overlapping ORF known as P3N‐PIPO was identified in the nine sequences.

For the remaining 15 samples subjected to NGS, between 2,329,452 and 4,234,476 raw reads were obtained, which after trimming resulted in between 2,323,748 and 4,232,646 reads per sample. De novo assembly of the reads confirmed the presence of CMV in the three samples which had tested positive for CMV using RT‐PCR (Ug90, 91 and 92), while in the remaining samples no contigs with identity to plant virus sequences were obtained after BLAST analysis (Table S2).

### 3.4 Evolutionary analysis

**FIGURE 3:**
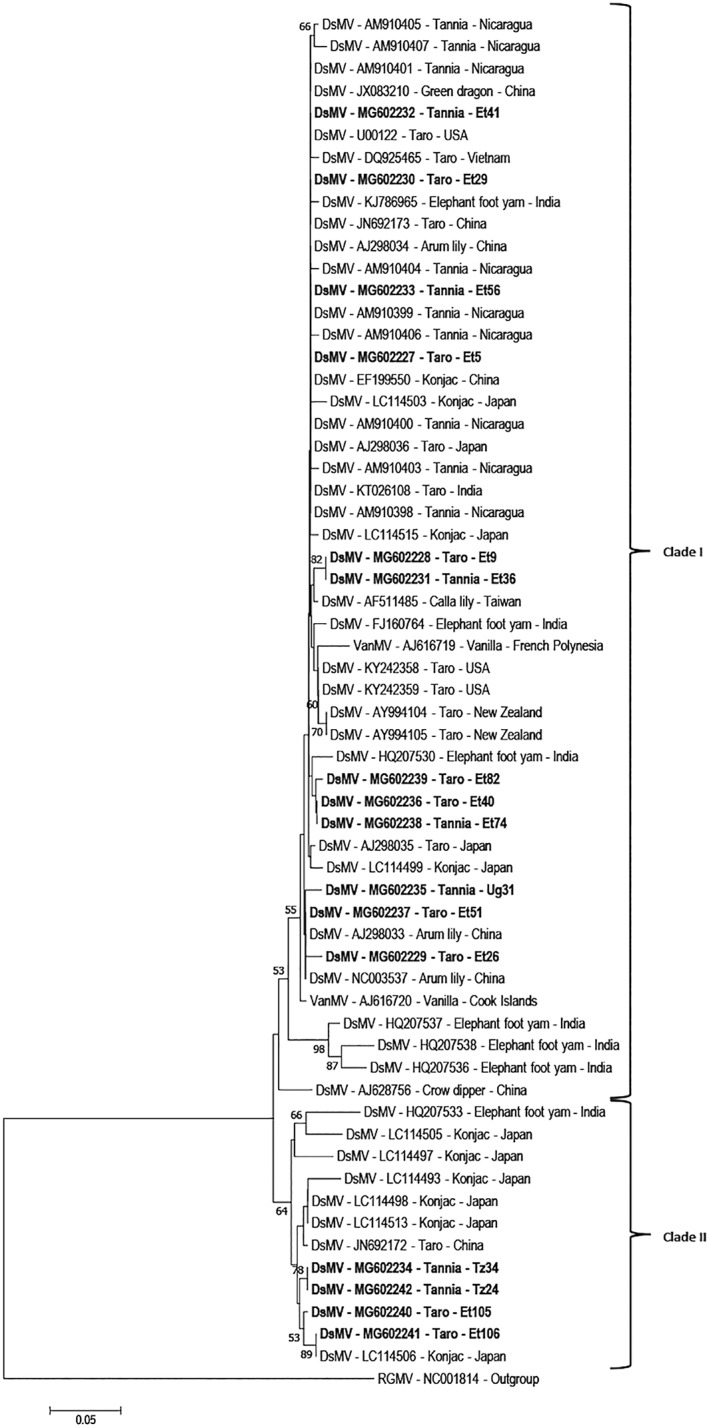
Phylogenetic analysis based on amino acid sequences of the core CP‐coding region of selected DsMV isolates. Phylogenetic tree generated using the Neighbour‐joining method with 1,000 bootstrap replications in MEGA7. The tree was rooted using RGMV, genus *Rymovirus*, family *Potyviridae* as an outgroup. Bootstrap values greater than 50% are shown. Taro (*Colocasia esculenta*), tannia (*Xanthosoma* sp.), elephant foot yam (*Amorphophallus paeoniifolius*), konjac (*Amorphophallus konjac*), arum lily (*Z*. *aethiopica*), calla lily (*Zantedeschia* sp.). DsMV, dasheen mosaic virus; VanMV, vanilla mosaic virus); RGMV, ryegrass mosaic virus

Phylogenetic analysis was carried out using the amino acid sequences of the core CP‐coding region from RT‐PCR amplicons from the 16 DsMV isolates sequenced from East Africa, together with published DsMV isolates and Ryegrass mosaic virus as an outgroup. The DsMV isolates grouped into two distinct clades with isolates from East Africa distributed across both clades (Figure 3). Twelve of the 16 isolates sequenced in this study grouped with ‘clade I’, including 11 of the isolates from Ethiopia infecting both taro and tannia, as well as Ug31 infecting tannia in Uganda. The 12 isolates did not cluster together, but instead were dispersed among sequences from a range of geographic locations and host plant species. The clustering in clade I was not representative of either host plant species or geographic origins which consists of DsMV isolates infecting a wide range of aroids from China, Japan, Taiwan, Nicaragua, USA, Vietnam and New Zealand together with DsMV isolates from Ethiopia and Uganda (Figure 3). The remaining four isolates from East Africa grouped together within the smaller ‘clade II’ together with isolates from China, India or Japan, mostly infecting konjac (Figure 3). This included two of the Ethiopian isolates (Et105 and Et106) together with the two Tanzanian isolates sequenced.

**FIGURE 4:**
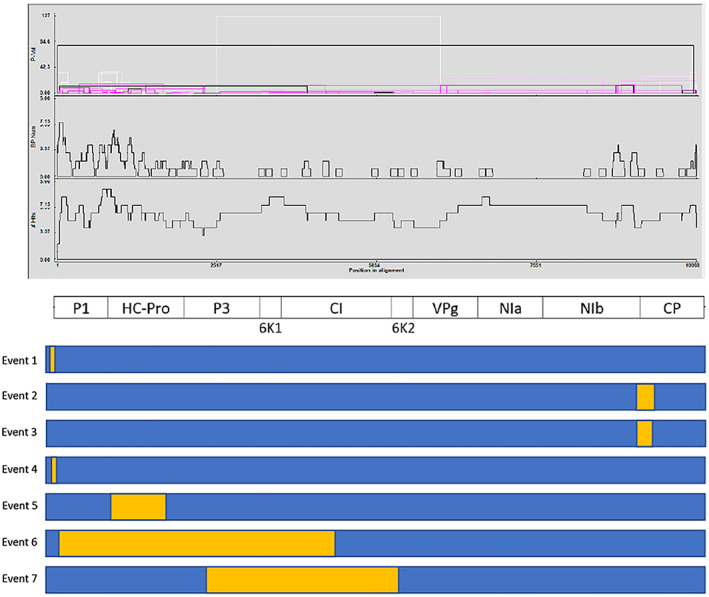
Recombination events detected using RDP4 for six previously characterised and nine new DsMV isolates based on their full‐length genome sequences. The top panel shows the distribution of hotspot sites in the DsMV genome. P‐Val: the minimum probability values associated with detected events; BP Num: Breakpoint number; #Hits: the number of events detected in particular regions of the alignment. Middle panel: Genomic organisation of DsMV. Bottom panel: pattern of recombination highlighted in yellow. The recombinant number corresponds with those in Table S3. DsMV, dasheen mosaic virus

Recombination analysis identified seven putative events between the 15 full‐length DsMV sequences analysed (Table S3; Figure 4). Recombination events were identified in several regions of the genome, with P1, HC‐Pro, P3 and NIb identified as the regions containing most breakpoints (Figure 4). Of the seven putative recombination events detected, four events were identified from East African sequences (events 1, 2, 5 and 6), including two events in Et5, one event in Et41 and one event in Tz34. Of the remaining three events (3, 4 and 7), one was identified in an isolate from the USA (KY242358), while the remaining two recombination events were in isolates from China (NC003537 and JX083210). Three of the four events identified in East African sequences involved only East African isolates as putative parents (events 1, 5 and 6). The putative recombination event in isolate Tz34 (event 2) included an isolate from the USA as a major putative parent. All the three recombination events in isolates from either China or the USA have an African isolate as one (event 3 and 7) or both (event 4) of the putative parents, while event 7 includes a putative minor parent from India (KT026108) (Table S3).

Analysis of the dN/dS substitution ratios for the individual coding regions of the 15 full‐length DsMV sequences revealed a moderate to high negative (purifying) selection pressure (dN/dS <1) for all coding regions (Figure S1A). These ratios vary between a very high purifying selection pressure of 0.04 in the 6K1 region to a relatively moderate purifying selection pressure of 0.54 in the P3 gene (Figure S1A). The HC‐Pro, 6K1, CI, 6K2, VPg, NIa and NIb genes are under high to very high negative selection pressure, whereas the P1, P3 and CP genes were found to be under a relatively moderate negative selection pressure.

PASC analysis based on the CP amino acid sequences of DsMV isolates from East Africa together with previously reported isolates from the NCBI database revealed similarity ranging between 88.3% and 100% (Table S4). Subsequently, PASC analysis based on the individual protein‐coding regions of the 15 available full‐length DsMV isolates were carried out both at the nucleotide and amino acid levels. When East African DsMV isolates were considered, amino acid similarities ranging from 90.4 to 100% were observed within the 6K1, CI, VPg and CP regions (Table S4). In contrast, amino acid similarities in the P1, P3 and 6K2 regions for East African isolates varied between 72 and 100%. Comparison of East African DsMV sequences with previously reported isolates from NCBI showed highest similarity (>90%) within the 6K1 and CI regions, while the P1 and P3 regions showed the lowest amino acid similarities (Table S4). PASC analysis based on nucleotide sequences revealed that the P1 region has the lowest level of identity (61.4%), while the remaining nine gene products showed more than 70% nucleotide sequence identity, either among the DsMV isolates from East Africa sequenced in this study or together with previously characterised sequences (Kidanemariam et al., 2018; Wang et al., 2017). Further comparison of DsMV full‐length nucleotide sequences using the SSE platform also identified the highest mean pairwise distances (both within and between geographic groups) within the P1 region, ranging from 0.25 to 0.33, followed by the P3 region and the NIB/CP junction (Figure S1B), consistent with the PASC analysis described previously.

## 4 DISCUSSION

Of the 392 samples collected from 25 regions in the four countries, a total of 91 (68 taro and 23 tannia) samples showed virus‐like symptoms similar to those associated with virus infection in a range of aroids (Elliott et al., 1997; Revill, Jackson, et al., 2005; Zettler et al., 1970). Symptomatic plants were found in 21 regions surveyed, with no virus‐like symptoms observed in Oromia in Ethiopia, Siaya in Kenya, or Budondo and Kignlu in Uganda (Table 2). No plants showed symptoms typical of bobone or alomae disease.

Of the 91 symptomatic samples, 45 samples from Ethiopia, Tanzania and Uganda showed symptoms such as feathery mottle, mosaic, leaf distortion, yellowing and/or stunting, which have been associated with DsMV infection (Figure 2a–k) (Nelson, 2008). Of these 45 symptomatic samples, 26 were confirmed to be infected with DsMV, including 23 samples from Ethiopia, one from Uganda and two from Tanzania. In addition, 13 asymptomatic plants from Ethiopia together with an asymptomatic sample from Tanzania (Tz24) also tested positive for DsMV. This phenomenon is consistent with previous studies and may occur as a consequence of seasonal effects or differences in symptom expression in different host plant species (Elliott et al., 1997; Nelson, 2008). Three tannia samples collected from a single site in Luuka region of Uganda which showed mosaic, mottling and vein‐chlorosis symptoms (Figure 2j and k) were found to be infected with CMV and not DsMV. This finding was confirmed by NGS analysis, with CMV the only virus detected in these three samples. A comprehensive analysis of the CMV isolates in these samples was reported previously (Kidanemariam et al., 2019). Although a range of other symptoms were observed in the samples collected in this study, no other samples tested positive for CMV, suggesting that asymptomatic infections of taro and tannia with CMV were not present in any of the samples collected and that symptoms observed on other samples were not associated with CMV infection. The survey findings suggest that, while DsMV is widespread in Ethiopia, being detected in ~21% of the 171 samples collected from the five regions surveyed, this is not the case in Uganda, Tanzania and Kenya. Six samples (four from Ethiopia and two from Uganda) with DsMV‐like symptoms tested negative for all the viruses. The yellowing and mosaic symptoms observed on these six samples might be caused by other factors such as senescence, pests, pesticide use or viruses other than DsMV, CMV or the two rhabdoviruses assayed.

Forty‐six samples showing symptoms such as leaf discolouration or yellowing, vein‐swelling or deformation, downward‐curling of the leaf blades, or stunting, tested negative for all the assayed viruses. These samples included 36 from taro and 10 from tannia and were collected from districts throughout the four countries surveyed. The symptoms observed on these samples may be caused by any one of several factors, such as nutritional deficiencies, as yet unidentified virus/es, or by other aroid‐infecting viruses for which testing was not carried out, such as viruses from the families *Reoviridae* and *Tospoviridae*. It is also possible that the plants were infected with sequence variants of DsMV, CMV, CBDaV or TaVCV whose diversity precluded their detection using the currently available primers. To investigate the possibility of novel viruses or isolates in the survey samples, eight samples with symptoms including leaf yellowing, vein clearing, and leaf deformation and four samples without symptoms were selected for NGS (Table S2). Interestingly, de novo assembly, followed by BLAST analysis, failed to identify any viruses in the selected samples. This result confirms that in some cases, the observed symptoms are probably not associated with viral infections.

In work associated with the current study (Kidanemariam, Sukal, et al., 2018), the same leaf samples were tested for badnaviruses using PCR and rolling circle amplification and full‐length sequences were characterised (Table 2). A high incidence and wide distribution of both TaBV and TaBCHV was determined, with at least one sample from every district testing positive; however, there was no clear association of either of these two viruses with symptoms. A comparison of the results of these two studies indicates that mixed infections of TaBV and DsMV can occur. Of the 36 and three samples from Ethiopia and Tanzania which tested positive for DsMV, 23 (63.8%) and two (66.7%) samples also tested positive for badnavirus, respectively (Table 2). However, the single sample from Uganda which tested positive for DsMV did not test positive for badnavirus (Table 2). Further work on the synergistic effects of mixed infections, compared to infection with either TaBV or DsMV alone, on the yield of taro plants is warranted. Interestingly, there were no mixed infections between the badnavirus, TaBCHV and DsMV.

Although partial NIb‐coding region sequences of DsMV isolates from Ethiopia are available (Kidanemariam, Macharia, et al., 2018), no complete genomic sequences of East African DsMV isolates have been reported before. Therefore, the complete genome sequences of nine East African isolates were determined and analyses were carried out to determine the evolutionary relationship of these and previously reported DsMV isolates. The genome organisation of the nine DsMV isolates was consistent with other previously characterised DsMV isolates. Phylogenetic analysis carried out using the core CP‐coding amino acid sequences was also consistent with previous reports, with DsMV isolates grouping into two distinct clades (Babu & Hegde, 2014; Wang et al., 2017). The separation of Ethiopian DsMV isolates across the two clades each containing isolates from different geographic locations including, China, Nicaragua, Taiwan, India, the USA and Japan suggests that the virus has most likely been introduced from different sources on multiple occasions. Similarly, the isolates sequenced in the present study from Uganda (clade I) and Tanzania (clade II) are members of distinct subgroups and most probably have distinct origins. The phylogenetic analysis also revealed no relationships between clades with respect to geographic origin or host plant among the DsMV isolates included in this study, which is also consistent with previous work (Wang et al., 2017).

Recombination contributes to the evolution of potyviruses and facilitates adaptation to new hosts or environments (Gagarinova, Babu, Strömvik, & Wang, 2008; Revers, Le Gall, Candresse, Le Romancer, & Dunez, 1996). Analysis of the nine full‐length genome sequences reported in the present study, together with the six previously published complete genome sequences of DsMV, revealed a high rate of recombination between virus isolates. This finding is consistent with the previous study conducted by Wang et al. (2017) where three recombination events were detected out of the six full‐length DsMV isolates available at that time. The analysis also revealed that some recombinant isolates are also either major or minor parents in other recombination events showing a high degree of recombination has occurred in DsMV. For instance, isolate Et5 was identified as a recombinant (events 1 and 5), and as a parent in the other recombination events (events 2 and 6) (Table S3). Similar patterns of recombination in potyviruses infecting sweet potato, where an isolate appeared as a recombinant in several events and at the same time was identified as a parent in other recombination events, were reported in Maina, Barbetti, Martin, Edwards, and Jones (2018).

The low dN/dS ratio (<1) across all genes of DsMV revealed the presence of a negative (purifying) selection pressure predicting a slow evolutionary rate. The HC‐Pro, 6K1, CI, 6K2, VPg, NIa and NIb genes showed the highest purifying selective pressure (0.04–0.12) while the P1, P3 and CP gene products of DsMV showed a relatively lower purifying selection pressure of 0.29, 0.54 and 0.52, respectively (Figure S1A). Variability in the levels of negative selection pressures is also typical of other members of the genus *Potyvirus* (Gibbs, Hajizadeh, Ohshima, & Jones, 2020; Nigam, LaTourrette, Souza, & Garcia‐Ruiz, 2019). Although gene products with critical roles in replication complexes and host–membrane interactions are under the highest selective pressures, proteins involved in translation, virion formation and vector transmission can be more variable and may assist these viruses in infecting a range of host plants in different agro‐ecological zones (Nigam et al., 2019). P1 and P3 both have hypervariable regions which contribute to host adaptation, pathogenicity and symptoms while CP variability influences aphid transmission and host range (Nigam et al., 2019).

This is the first comprehensive survey carried out in East Africa to identify and characterise RNA viruses infecting taro and tannia in the region. The findings from this study will assist farmers and national agricultural research services in the region to make informed decisions regarding the acquisition and dissemination of edible aroids. The high prevalence of DsMV in Ethiopia should necessitate further work on the yield losses caused by this virus and the identification of tolerant/resistant cultivars. The establishment of virus‐indexed tissue culture nurseries within East Africa will play a key role in the production and distribution of virus‐free farmer‐preferred edible aroid cultivars in the region. The full‐length and partial genome sequences of DsMV isolates generated in this study from East Africa are expected to facilitate further studies on the detection, population structure and evolution of aroid viruses worldwide. The collection of field samples from this work will be preserved at the BecA‐ILRI Hub and will be available for further virus testing, if and when additional diagnostic assays become available. This may shed light on the cause of the symptoms displayed on some plants which tested negative in the current work.

## CONFLICT OF INTEREST

The authors declare they have no conflict of interest.

## Supporting information


**SUPPLEMENTARY FIGURE 1** A) Selection pressure across the protein‐coding regions of the 15 full‐length DsMV isolates; B) Distance plot of DsMV full‐length nucleotide sequences.

**SUPPLEMENTARY TABLE 1** Summary of Next Generation Sequencing (NGS) and Sanger sequencing data for DsMV‐infected samples selected for sequencing.

**SUPPLEMENTARY TABLE 2** Summary of Next Generation Sequencing (NGS) data for additional samples selected for sequencing.

**SUPPLEMENTARY TABLE 3** Summary of the recombination events identified by RDP4 between all available full‐length DsMV genomic sequences.

**SUPPLEMENTARY TABLE 4** Summary of PASC analysis of nucleotide (nt) and amino acid (aa) sequences for each of the different protein‐coding regions of all available full‐length DsMV genomic sequences.
