# Supplementary figures and images for: Incidence of RNA viruses infecting taro and tannia in East Africa and molecular characterisation of dasheen mosaic virus isolates
Source: Ann Appl Biol. 2021 Sep 7;180(2):211–23. doi: 10.1111/aab.12725 (PMC9293211; doi:10.1111/aab.12725)

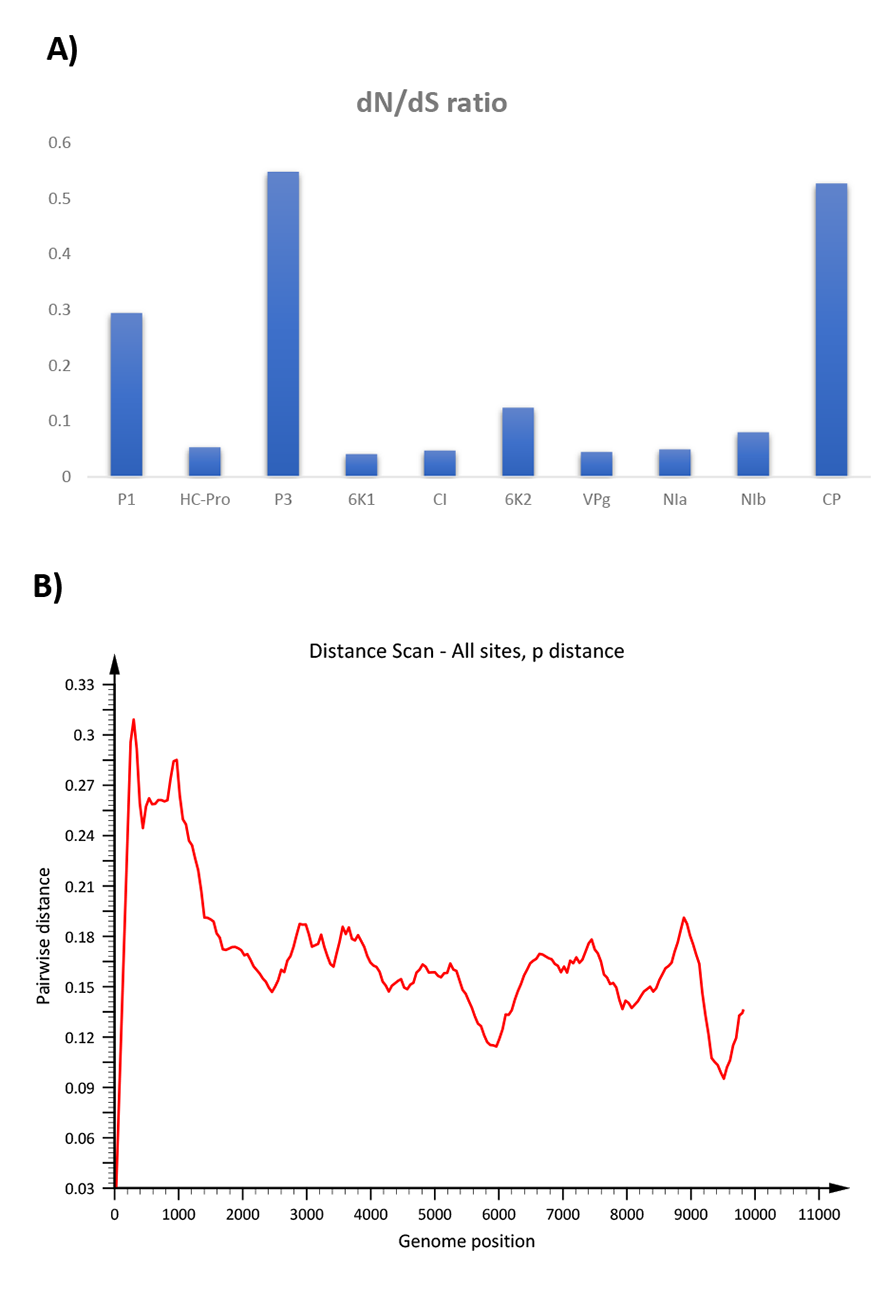

Supplement: Supplementary file 1 — SUPPLEMENTARY FIGURE 1 A) Selection pressure across the protein‐coding regions of the 15 full‐length DsMV isolates; B) Distance plot of DsMV full‐length nucleotide sequences. [file AAB-180-211-s003.tif]
