# Supplementary material for: Incidence of RNA viruses infecting taro and tannia in East Africa and molecular characterisation of dasheen mosaic virus isolates
Source: Ann Appl Biol. 2021 Sep 7;180(2):211–23. doi: 10.1111/aab.12725 (PMC9293211; doi:10.1111/aab.12725)
Supplement: Supplementary file 2 — SUPPLEMENTARY TABLE 1 Summary of Next Generation Sequencing (NGS) and Sanger sequencing data for DsMV‐infected samples selected for sequencing. [file AAB-180-211-s002.docx]

**Supplementary Table 1**. Summary of Next Generation Sequencing (NGS) and Sanger sequencing data for DsMV-infected samples selected for sequencing.

| Sequencing technology | Sample ID | No. of raw reads obtained | No. of reads  after trimming | Ref. seq. used for mapping | No. of reads mapped to  ref. seq. | Final sequence  length | NCBI accession  no. |
| --- | --- | --- | --- | --- | --- | --- | --- |
| NGS | Et5 | 1,266,579 | 1,091,125 | NC003537 | 398,788 | 10,026 | MG602227 |
|  | Et9 | 1,396,901 | 1,199,687 |  | 401,698 | 10,026 | MG602228 |
|  | Et26 | 1,950,018 | 1,002,781 |  | 251,003 | 10,023 | MG602229 |
|  | Et29 | 1,823,943 | 983,743 |  | 212,988 | 10,025 | MG602230 |
|  | Et36 | 1,164,718 | 971,694 |  | 202,476 | 10,028 | MG602231 |
|  | Et41 | 2,855,123 | 1,912,801 |  | 339,528 | 10,027 | MG602232 |
|  | Et56 | 2,435,168 | 2,296,872 |  | 426,072 | 10,027 | MG602233 |
|  | Tz34 | 2,046,991 | 1,895,667 |  | 387,600 | 10,028 | MG602234 |
|  | Ug31 | 1,450,893 | 1,139,951 |  | 348,167 | 10,018 | MG602235 |
| Sanger | Et40 |  |  |  |  |  | MG602236 |
|  | Et51 |  |  |  |  |  | MG602237 |
|  | Et74 |  |  |  |  |  | MG602238 |
|  | Et82 |  |  |  |  |  | MG602239 |
|  | Et105 |  |  |  |  |  | MG602240 |
|  | Et106 |  |  |  |  |  | MG602241 |
|  | Tz24 |  |  |  |  |  | MG602242 |
