# Supplementary material for: Incidence of RNA viruses infecting taro and tannia in East Africa and molecular characterisation of dasheen mosaic virus isolates
Source: Ann Appl Biol. 2021 Sep 7;180(2):211–23. doi: 10.1111/aab.12725 (PMC9293211; doi:10.1111/aab.12725)
Supplement: Supplementary file 3 — SUPPLEMENTARY TABLE 2 Summary of Next Generation Sequencing (NGS) data for additional samples selected for sequencing. [file AAB-180-211-s001.docx]

**Supplementary Table 2**. Summary of Next Generation Sequencing (NGS) data for additional samples selected for sequencing.

| Sample | Country | Reason for NGS | No. of raw reads | No. of trimmed reads | Virus/es identified |
| --- | --- | --- | --- | --- | --- |
| Et39 | Ethiopia | Virus-like symptoms | 2,329,452 | 2,323,748 | No virus identified |
| Ke11 | Kenya | Asymptomatic | 3,088,490 | 3,087,248 | No virus identified |
| Ke23 |  | Virus-like symptoms | 3,037,864 | 3,036,850 | No virus identified |
| Ke28 |  | Virus-like symptoms | 3,201,438 | 3,200,118 | No virus identified |
| Ke63 |  | Asymptomatic | 2,346,502 | 2,343,942 | No virus identified |
| Tz2 | Tanzania | Asymptomatic | 3,247,842 | 3,245,622 | No virus identified |
| Tz22 |  | Virus-like symptoms | 2,690,596 | 2,690,022 | No virus identified |
| Tz24 |  | Asymptomatic | 3,226,322 | 3,224,802 | No virus identified |
| Tz25 |  | Virus-like symptoms | 2,729,590 | 2,728,634 | No virus identified |
| Ug35 | Uganda | Virus-like symptoms | 3,074,052 | 3,072,054 | No virus identified |
| Ug44 |  | Virus-like symptoms | 4,234,476 | 4,232,646 | No virus identified |
| Ug70 |  | Virus-like symptoms | 3,640,262 | 3,639,800 | No virus identified |
| Ug90 |  | Virus-like symptoms | 3,452,634 | 3,294,042 | CMV full length |
| Ug91 |  | Virus-like symptoms | 3,629,228 | 3,108,688 | CMV full length |
| Ug92 |  | Virus-like symptoms | 2,893,680 | 2,586,760 | CMV full length |
