# Supplementary material for: Incidence of RNA viruses infecting taro and tannia in East Africa and molecular characterisation of dasheen mosaic virus isolates
Source: Ann Appl Biol. 2021 Sep 7;180(2):211–23. doi: 10.1111/aab.12725 (PMC9293211; doi:10.1111/aab.12725)
Supplement: Supplementary file 4 — SUPPLEMENTARY TABLE 3 Summary of the recombination events identified by RDP4 between all available full‐length DsMV genomic sequences. [file AAB-180-211-s004.docx]

**Supplementary Table 3.** Summary of the recombination events identified by RDP4 between all available full-length DsMV genomic sequences.

|  | Breakpoint Positions^a^ | | Recombinant Sequence | | | Detection Method^d^ | | | | | | |
| --- | --- | --- | --- | --- | --- | --- | --- | --- | --- | --- | --- | --- |
| Recombination Events | Begin | End | Recombinant Seq | Minor Parental Seq^b^ | Major Parental Seq^c^ | RDP | GENECONV | Bootscan | MaxChi | Chimaera | SiScan | 3SEQ |
| 1 | 70 | 168 | Et5 (MG602227) | Et26 (MG602229) | Et9 (MG602228) | 9.18E-16 | 1.29E-18 | 3.08E-13 | 7.42E-09 | 1.07E-07 | 7.64E-13 | 1.43E-07 |
| 2 | 8752 | 9070 | Tz34 (MG602234) | Et5 (MG602227) | KY242358 | 3.42E-11 | 1.99E-09 | NS | 1.07E-04 | 3.03E-07 | NS | 3.68E-02 |
| 3 | 8722 | 8997 | KY242358 | JX083210 | Tz34 (MG602234) | 5.14E-13 | 2.87E-10 | 3.28E-11 | 1.92E-07 | 9.86E-07 | 3.28E-13 | 1.12E-05 |
| 4 | 82 | 149 | NC003537 | Et56 (MG602233) | Et26 (MG602229) | 1.91E-05 | 5.64E-09 | NS | 8.66E-05 | 1.08E-02 | 1.59E-06 | NS |
| 5 | 1016 | 1809 | Et5 (MG602227) | Et36 (MG602231) | Et41 (MG602232) | 1.53E-07 | NS | 1.48E-04 | 6.49E-04 | 7.51E-04 | 2.77E-08 | 4.35E-02 |
| 6 | 280 | 4377 | Et41 (MG602232) | Et5 (MG602227) | Et29 (MG602230) | 4.01E-02 | NS | NS | 1.02E-02 | 5.11E-05 | 7.29E-15 | 5.05E-14 |
| 7 | 2408 | 5287 | JX083210 | KT026108 | Et26 (MG602229) | NS | NS | NS | 3.20E-02 | 2.05E-04 | 2.67E-02 | 1.52E-03 |

^a^ Position refers to the major parental sequence indicated

^b^ Parent strain contributing the smaller fraction of the sequence

^c^ Parent strain contributing the larger fraction of the sequence

^d^ NS: No significant P value was obtained using this method
