# Supplementary material for: Incidence of RNA viruses infecting taro and tannia in East Africa and molecular characterisation of dasheen mosaic virus isolates
Source: Ann Appl Biol. 2021 Sep 7;180(2):211–23. doi: 10.1111/aab.12725 (PMC9293211; doi:10.1111/aab.12725)
Supplement: Supplementary file 5 — SUPPLEMENTARY TABLE 4 Summary of PASC analysis of nucleotide (nt) and amino acid (aa) sequences for each of the different protein‐coding regions of all available full‐length DsMV genomic sequences. [file AAB-180-211-s005.docx]

**Supplementary Table 4**. Summary of PASC analysis of nucleotide (nt) and amino acid (aa) sequences for each of the different protein-coding regions of all available full-length DsMV genomic sequences.

|  | East African | | Worldwide | |
| --- | --- | --- | --- | --- |
|  | nt (%) | aa (%) | nt (%) | aa (%) |
| P1 | 72.3–100 | 72–100 | 61.4–100 | 60–100 |
| HC-Pro | 76–100 | 89.7–100 | 76–100 | 89.5–100 |
| P3 | 72–99.8 | 72–99.7 | 70.2–99.8 | 69–99.7 |
| 6K1 | 75–100 | 90.4–100 | 73.7–100 | 90.4–100 |
| CI | 78.9–99.9 | 91.3–100 | 78.8–99.9 | 91.2–100 |
| 6K2 | 75.4–100 | 79.2–100 | 75.4–100 | 79.2–100 |
| VPg | 79.1–100 | 95.3–100 | 78–100 | 62.7–100 |
| NIa | 78.3–100 | 87.6–100 | 77.8–100 | 87.6–100 |
| NIb | 79.8–100 | 88.2–100 | 78.1–100 | 86–100 |
| CP | 83.2–99.8 | 92–99.7 | 77–99.8 | 79.6–99.7 |
| Full-length | 75–100 | 86.4–99.8 | 75.4–99.8 | 84.6–99.8 |
